# Strongly Inhibited Spontaneous Emission of PbS Quantum Dots Covalently Bound to 3D Silicon Photonic Band Gap Crystals

**DOI:** 10.1021/acs.jpcc.4c01541

**Published:** 2024-05-28

**Authors:** Andreas S. Schulz, Marek Kozoň, G. Julius Vancso, Jurriaan Huskens, Willem L. Vos

**Affiliations:** † Complex Photonic Systems (COPS), MESA+ Institute, University of Twente, P.O. Box 217, 7500 AE Enschede, The Netherlands; ‡ Molecular Nanofabrication (MNF), MESA+ Institute, University of Twente, P.O. Box 217, 7500 AE Enschede, The Netherlands; § Materials Science and Technology of Polymers (MTP), MESA+ Institute, University of Twente, P.O. Box 217, 7500 AE Enschede, The Netherlands; ∥ Mathematics of Computational Science (MACS), MESA+ Institute, University of Twente, P.O. Box 217, 7500 AE Enschede, The Netherlands; ⊥ Sustainable Polymer Chemistry (SPC), MESA+ Institute, University of Twente, P.O. Box 217, 7500 AE Enschede, The Netherlands

## Abstract

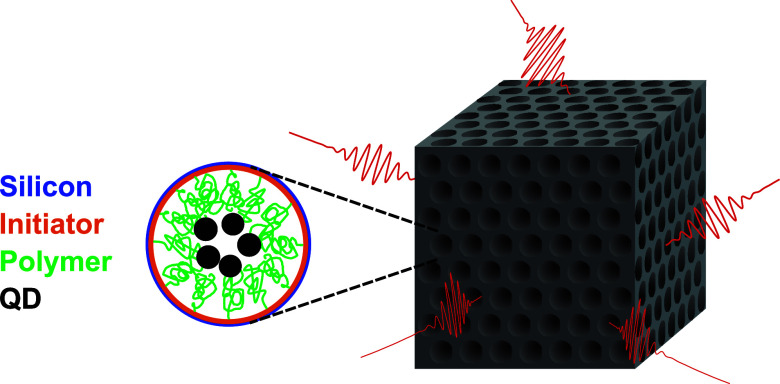

We present an optical study of the spontaneous emission of lead sulfide (PbS) nanocrystal quantum dots in 3D photonic band gap crystals made from silicon. The nanocrystals emit in the near-infrared range to be compatible with 3D silicon nanophotonics. The nanocrystals are covalently bound to polymer brush layers that are grafted from the Si–air interfaces inside the nanostructure by using surface-initiated atom transfer radical polymerization. The presence and position of the quantum dots were previously characterized by synchrotron X-ray fluorescence tomography. We report both continuous wave emission spectra and time-resolved, time-correlated single photon counting. In time-resolved measurements, we observe that the total emission rate greatly increases when the quantum dots are transferred from suspension to the silicon nanostructures, likely due to quenching (or increased nonradiative decay) that is tentatively attributed to the presence of Cu catalysts during the synthesis. In this regime, continuous wave emission spectra are known to be proportional to the radiative rate and thus to the local density of states. In spectra normalized to those taken on flat silicon outside the crystals, we observe a broad and deep stop band that we attribute to a 3D photonic band gap with a relative bandwidth of up to 26%. The shapes of the relative emission spectra match well with the theoretical density of states spectra calculated with plane-wave expansion. The observed inhibition is 4–30 times, similar to previously reported record inhibitions, yet for coincidental reasons. Our results are relevant to applications in photochemistry, sensing, photovoltaics, and efficient miniature light sources.

## Introduction

The intriguing opportunity to control the properties of matter via light lies at the heart of quantum optics and cavity quantum electrodynamics (cQED). A famous example is the control of the radiative rate of an elementary quantum emitter such as excited atoms, ions, molecules, 2D materials, or quantum dots.^1^ Such control is essential for applications ranging from miniature lasers and light-emitting diodes,^2,3^ to single-photon sources for quantum information,^4−6^ solar energy harvesting,^7,8^ to photocatalysis and photochemistry,^9−11^ and sensing.^12,13^

**Figure 1:**
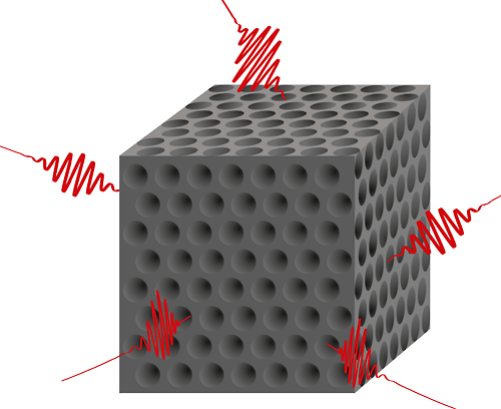
Cartoon of a finite 3D photonic crystal in free space. Since the crystal has a complete 3D photonic band gap, ubiquitous vacuum fluctuations incident on the crystal’s surface (shown as red wavelets) are forbidden from entering and are thus reflected from the crystal’s surfaces. Hence, an excited two-level quantum system (atom, ion, molecule, or quantum dot) embedded inside the crystal is shielded from the fluctuations and cannot decay by spontaneously emitting a photon. Thus, the excited state becomes more stable.

When the emitter’s properties are in the quantum regime, as is the case with nanoemitters in the optical range, a major role is played by the fluctuations of the quantized electromagnetic fields, so-called vacuum fluctuations.^14,15^Figure 1 schematically illustrates such fluctuations that exist everywhere, even when there are zero photons. By surrounding an emitter by a suitably tailored dielectric nanostructured environment, the vacuum fluctuations are controlled; the most radical control is offered by photonic band gap crystals, as discussed in this paper. Inside such nanostructures, all light waves (with many photons) and hence all vacuum fluctuations (with zero photons) are forbidden by interference (since both are solutions of Maxwell’s equations) and are thus expelled, as illustrated in Figure 1.

Long after the pioneering realization by Purcell that an emitter’s environment such as a cavity controls the emission rate,^16^ emission control has become one of the main drivers of the field of nanophotonics.^17−21^ Following seminal work by Bykov and by Yablonovitch,^1,2^ emission control was first studied on periodic photonic crystals^22−32^ and recently has even been extended to 3D circuits^33^ and chiral emission.^34^ Emission control has also successfully been pursued with many different nanophotonic systems and many different quantum emitters, such as atoms and dye molecules in Fabry–Pérot microcavities,^35,36^ quantum dots in pillar microcavities^5,37^ and in disordered photonic materials,^38^ ions in whispering gallery-mode microspheres,^39−41^ dye molecules in plasmonic nanocavities and on nanoantennae,^42−46^ dye molecules in metamaterials,^47,48^ and diamond and perovskite nanocrystals in photonic crystals.^49,50^

**Figure 2:**
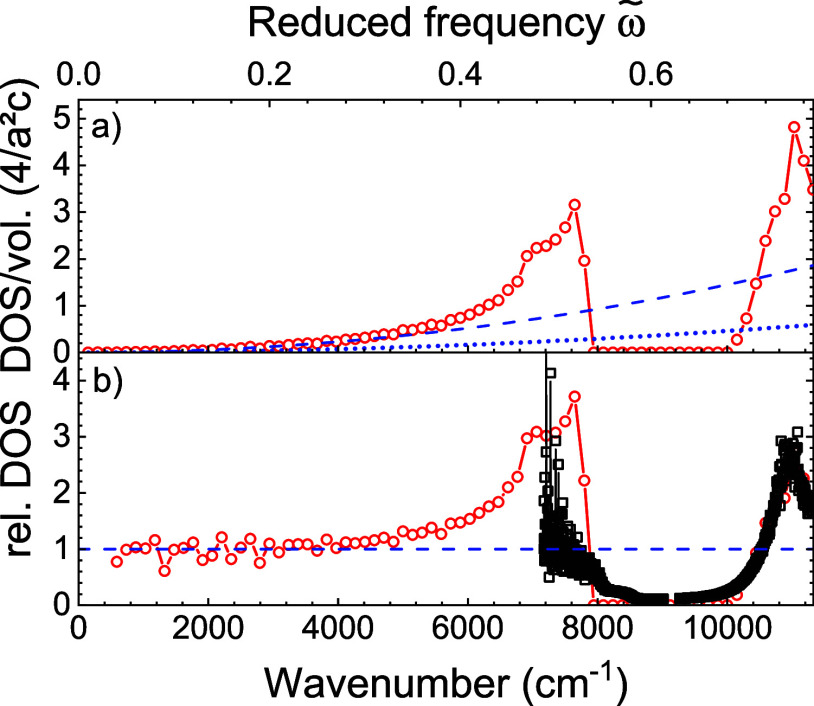
(a) DOS computed (red) for an inverse woodpile photonic band gap crystal from silicon (ε = 11.7) with pores with a reduced radius *R*/*a* = 0.252. Experiment results are shown for comparison (black squares). The theoretical DOS of a homogeneous medium with the same effective refractive index as the crystal (blue dashed line) and of free space (blue dotted curve) is shown. (b) Relative DOS equal to DOS of the crystal over DOS of the effective homogeneous medium. Black squares show our main result, namely the measured intensity of quantum dots inside a 3D silicon photonic crystal normalized to the intensity of similar quantum dots on a silicon surface, and reveal a broad band gap with strong inhibition.

**Figure 3:**
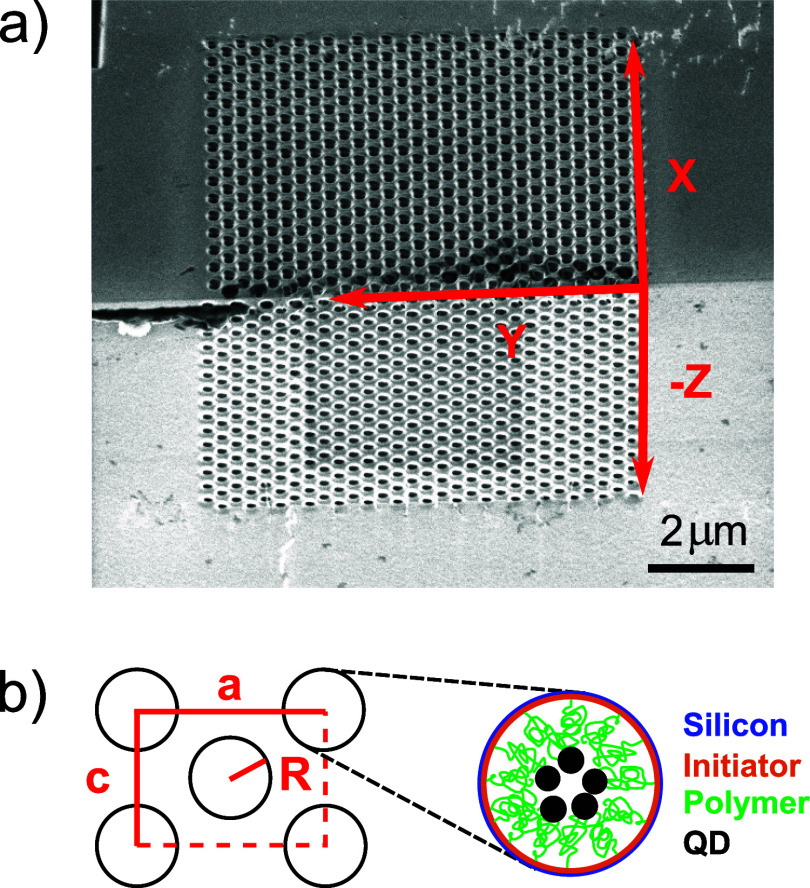
(a) Scanning electron micrograph of a 3D photonic band gap crystal viewed from 45° on the edge of the silicon beam showing the *XY* (top) and the *Y*Z surfaces (bottom); the scale bar indicates 2 μm. (b) View along the pores showing the lattice parameters *a*, *c* (with ) and pore radius *R*. Zoomed-in cross section of one pore with targeted polymer surface chemistry: ATRP initiator layer (orange), polymer chains forming brushes (green), and covalently attached PbS quantum dots (black) on top of silicon (blue).

Previously, our group studied the influence of a 3D photonic band gap on the emission of nanocrystalline quantum dots that were infiltrated as a liquid suspension.^27,31,51^ Hence, there was no control on the placement of the internal emitters, and the main question of “where the emitters reside inside the photonic crystal nanostructure” was addressed indirectly. Therefore, we have embarked on a journey to position emitters at targeted locations in space; moreover, we sought to exploit an independent probe to determine the positions of the emitters. Recently, we reported the targeted positioning of nanocrystalline quantum dots inside 3D silicon photonic band gap crystals by means of polymer brush layers that are grafted to the Si–air interfaces using surface-initiated atom transfer radical polymerization (SI-ATRP).^52,53^ The crystals have the inverse woodpile structure that consists of 2 perpendicular sets of crossing pores (see also Figure 3).^54^ We performed high-resolution synchrotron X-ray fluorescence tomography at a high 17 keV photon energy to obtain large penetration depths and efficient excitation of the elements of interest, and unequivocally observed that the quantum dots reside along the axes of the pores.^55^ In this paper, we present a detailed optical study of the quantum dots inside the 3D Si photonic band gap crystals and collect emission spectra and excited-state lifetimes. We observe that broad band strongly inhibited emission inside the 3D band gap in agreement with theory, as well as enhanced emission above the band gap, as shown in Figure 2b.

## Theory

It is well-known in quantum optics and cavity quantum electrodynamics that in the weak-coupling approximation^56^ (or Wigner–Weisskopf approximation^57^) spontaneous emission of an excited quantum emitter is precisely described by Fermi’s golden rule.^58^ In a modern reformulation,^18,21,59^ the radiative decay rate is linearly proportional to the local density of optical states (LDOS), a classical property that gives the density of vacuum fluctuations and thus the amount of vacuum noise experienced by a qubit.^60^ From the perspective of quantum electrodynamics, vacuum fluctuations stimulate an excited quantum system to decay from its excited state, thereby impeding the quantum functionality that is often available with excited states.^a^ The LDOS not only controls spontaneous emission and blackbody radiation but also plays a role in van der Waals and Casimir dispersion forces between nanoparticles and in Förster resonance energy transfer (FRET) between different emitters.^61^

Expressed in words, the LDOS counts the number of electromagnetic field states available for emission, where each state is weighted by its strength at the emitter’s position **r**_0_ and the field is projected along the emitter’s dipole axis.^21,59,62^ When the values are averaged over position and orientation, we obtain the density of optical states.

Since the Bragg length of our photonic band gap crystal (_Br_ = 300 nm^63^) is much less than both the size of the unit cell (*a* = 680 nm) and the size of the crystals (*L* = 10 μm), photons emitted by the quantum dots scatter multiple times before being collected, see also ref (25). Therefore, the collected photons are emitted by an ensemble of quantum dots at different positions inside a unit cell and with all orientations of the transition dipole moment. Hence, a suitable interpretation of the experimental observations corresponds to averaging the LDOS over orientations and positions, which is equal to the density of states (DOS).^21^

In Figure 2, the theoretical DOS for an inverse woodpile crystal with pores of reduced radius *R*/*a* = 0.252 is compared with experimental measurements. Figure 2a shows the frequency dependence of the DOS that *grosso modo* increases quadratically with frequency.^b^ In the reduced frequency range^c^ ω̃≡a/λ = 0.54 to 0.68 (with *a* the lattice parameter and λ the wavelength) the DOS is completely inhibited,^59,64−70^ which defines the complete 3D photonic band gap. In the photonic band gap, the vacuum fluctuation wavelets bounce off the crystal’s external surface, as portrayed in Figure 1. Since the total number of states is conserved, the DOS is enhanced outside the band gap. For comparison, the DOS of a homogeneous medium with the same effective refractive index as the crystal exhibits similar behavior in the low-frequency range to about ω̃ = 0.25, before further increasing quadratically. The DOS of free space is also quadratic, albeit much lower overall than in the effective medium since the DOS is proportional to the (effective) refractive index cubed.^21^

Figure 2b shows the photonic crystal’s relative DOS that is normalized to the DOS of the effective homogeneous medium. This normalization is notably useful for two main reasons: First, it is insightful to remove the parabolic dependence, so that unmodified DOS corresponds to a relative DOS equal to one and enhancements and inhibitions correspond to a relative DOS >1 and <1, respectively. Second, such a ratio is a model of experiments as in this paper, where we normalize the intensity collected from quantum emitters inside a photonic crystal to the intensity of similar emitters in a reference situation without a band gap. By such a procedure, we normalize the specific spectrum of the emitters in order to focus on the crystal properties. Note that similar procedures were used in previous work, where the effect of band gap crystals was studied on laser dye molecules and quantum wells.^25,26^ The black squares show the main result of this study, namely, the measurements of one of our 3D silicon photonic band gap crystals with quantum dots as internal quantum emitters. We find a broad range of strongly inhibited emission between about 8000 and 10,400 cm^–1^, characteristic of a 3D photonic band gap with a relative bandwidth (width over center frequency) of about 26%, which matches well with the theoretical relative DOS.

## Methods

### 3D Silicon Photonic Crystals

We study 3D photonic band gap crystals with the diamond-like inverse woodpile structure that consist of two perpendicular arrays of interpenetrating pores.^54^ Our 3D crystals are made from silicon and fabricated with a CMOS-compatible process that is described in detail in refs (71−73). In brief, we use Si beams (0.5 × 0.5 × 10 mm^3^) as substrates that are chemically etched to obtain perpendicular crystal surfaces. A thin layer of chromium (50 nm) serves as a hard mask that is deposited on two adjacent surfaces of such a Si beam. Via focused-ion beam writing with Ga ions, we define apertures in an etch mask in a single step on both faces into the chromium mask layer. We use deep reactive ion etching using the Bosch process to etch two perpendicular arrays of deep nanopores through the mask apertures into the silicon.

Figure 3 shows a SEM image of a successfully etched 3D photonic band gap crystal. The pores that enter into the XY surface (top) and into the *YZ* surface (bottom) mutually cross inside the Si beam and thereby form the 3D tetragonal inverse woodpile structure^74^ with a width of about 10 μm that has a broad 3D photonic band gap.^54,75,76^ The crystals are designed to have a lattice parameter of *a* = 680 nm and pore diameters of *d* = 260 or 320 nm. The pore diameters correspond to reduced radii of *R*/*a* = 0.191 and 0.235. From Figure 2, it is apparent that in this range of pore radii the 3D photonic band gap occurs between 8000 and 10,000 cm^–1^, in the near-infrared spectral range, see refs (75 and 76).

### Quantum Dots Positioned by Surface Chemistry

As internal emitters, we choose near-infrared-emitting lead sulfide quantum dots whose emission band includes telecom bands and is compatible with the transparency range of silicon below the electronic band gap at 1.1 eV corresponding to wavelengths longer than 1100 nm (see, e.g., ref (8)). In addition, quantum dots were chosen to have their emission spectrum overlap with the 3D photonic band gap as shown in Figure 2.

**Figure 4:**
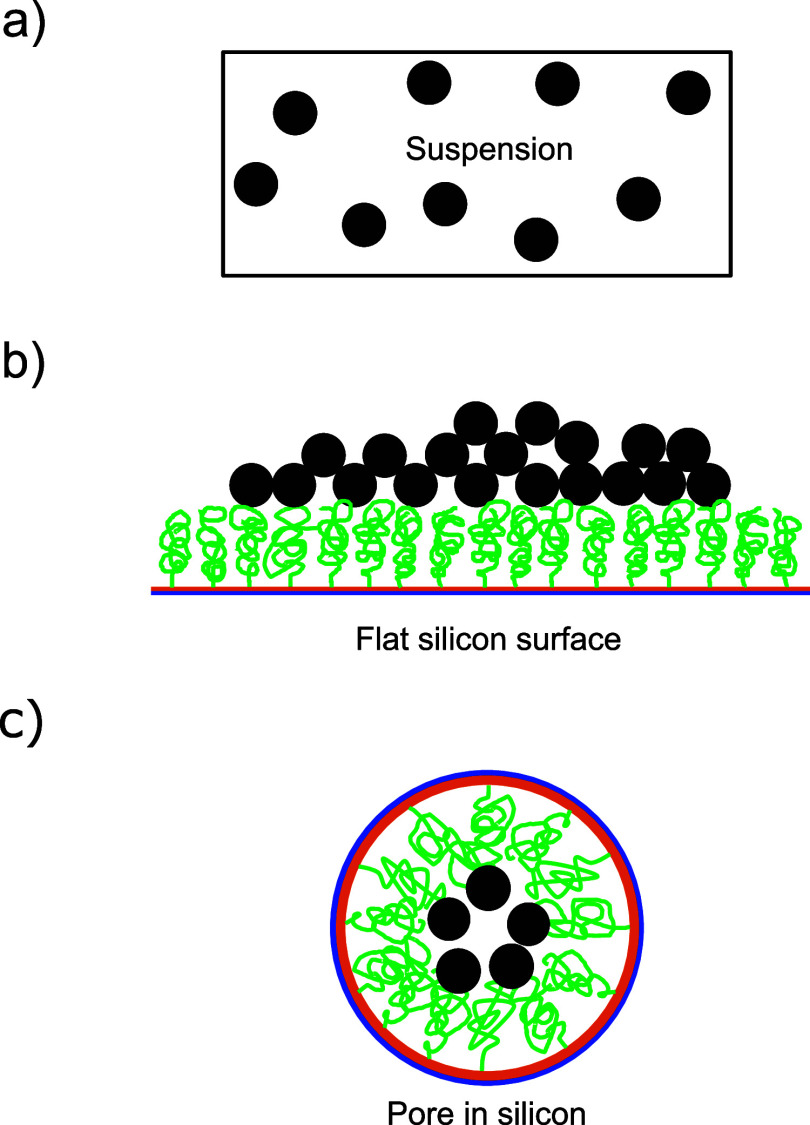
Schematic of how quantum dots are likely located in the three different samples studied here. (a) In suspension, the dots have a low density and are well-separated. (b) On a flat Si substrate, the quantum dots have a high areal density and are likely clustered or aggregated. (c) In a cross section of a crystal pore, the qunatum dots have a medium high areal density and are separated thanks to the targeted brush polymer surface chemistry.^55^

We analyze three different arrangements of quantum dots as depicted in Figure 4: in water suspension, attached to the flat silicon surface, and inside a 3D photonic band gap crystal.

To position the quantum dots inside the crystals, a thin polymer brush layer was grafted inside the pores to attach lead sulfide quantum dots, as described in detail in ref (55). The inorganic lead sulfide core of the quantum dots is covered by a poly(ethylene glycol)-amine (PEG-NH_2_) ligand that is used to couple to the polymer layer on the silicon photonic crystal, see Supporting Information, Scheme S1. Notably, in the process Cu catalysts are used. Since the quantum dot nanocrystals are much smaller than the photonic crystal pores, it is reasonable to assume that the dots do not have a preferred orientation and that their attachment to the brushes is random.

### Optical Setup

The optical microscope setup to collect both spectrally resolved and time-resolved spontaneous emission from the near-infrared quantum dots inside the 3D photonic band gap is described in detail in the Supporting Information. We excite the near-infrared-emitting quantum dots with short pulses from a pulsed diode laser that emits at λ = 690 nm (or 14,493 cm^–1^), with a narrow bandwidth less than 1 nm, i.e., far above the photonic band gap, where the excitation light is multiply scattered.^77^ For time-resolved measurements, the sample is excited from the *YZ* surface by focusing the incident laser beam with an objective (numerical aperture of NA = 0.12). Light emitted by quantum dots in the photonic crystal is collected from the *XY* face by a high-NA objective (NA = 0.72) and integrated for 600 s. In many collected spectra, a narrow range around 9100 cm^–1^ is absent since the involved diode array detector has a number of unresponsive (“dead”) pixels.

### Photonic DOS Computations

**Figure 5:**
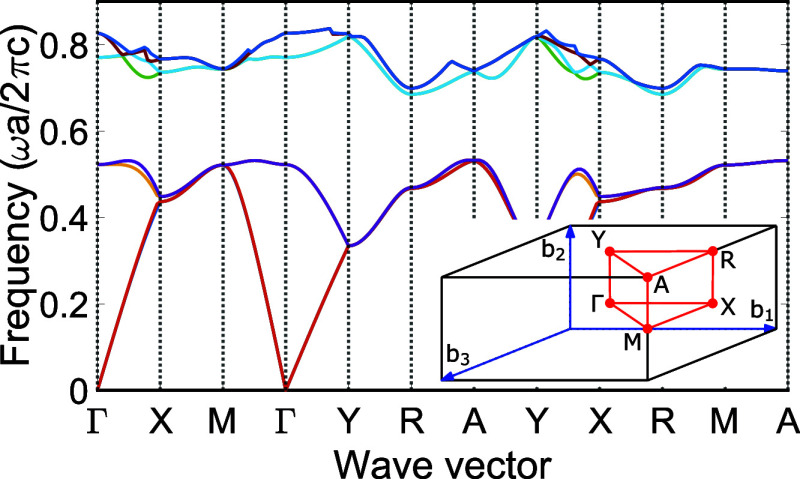
Band structure of the 3D inverse woodpile photonic band gap crystal with a reduced pore radius *R*/*a* = 0.252. The inset shows the Brillouin zone of the tetragonal unit cell with marked high-symmetry points and the tetragonal basis vectors **b**_**1**_, **b**_**2**_, and **b**_**3**_.

To compute the DOS of the 3D photonic band gap crystals, we have used the plane-wave expansion method,^78,79^ implemented in the well-known MPB package.^80^ At each wave vector **k**, the band frequencies ω̃(k) are computed. Figure 5 shows a resulting photonic band structure, in other words, the band frequencies **k** for trajectories in wave vector space between conventional high-symmetry points in the Brillouin zone, the latter being shown in the inset, see also ref (63). Around a reduced frequency of 0.6, a broad 3D photonic band gap is apparent, that gives rise to the DOS gap in Figure 2.

By sampling over all points within the Brillouin zone, we obtain the DOS  at a given frequency ω̃ as^78^
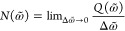
1with  being the number of states with frequencies in the interval . In the numerical case, frequency is discretized into bins of size Δω̃ and  becomes the number of states ω̃(*k*) found in each bin. The noise-like features in Figure 2b are well-known due to binning in frequency space to count modes.^69,80^ The spatial resolution of the employed tetragonal unit cell is 48 × 68 × 48, corresponding to a cutoff of *N* = 156,672 plane waves and the *k*-space resolution is 32 × 32 × 32 within the Brillouin zone, which is found in previous studies to be a suitable compromise between accuracy and computational time.^81^

## Results

### Emission Spectra

**Figure 6:**
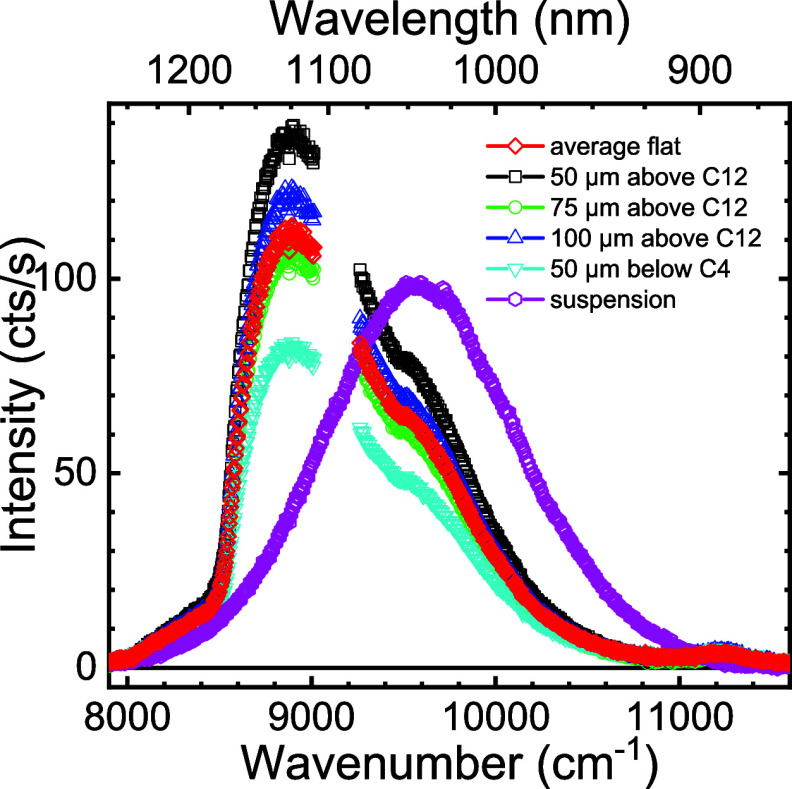
Emission spectrum of the PbS quantum dots in suspension is shown as magenta hexagons. The emission spectra of the quantum dots at four locations on the flat silicon surface on silicon beam 1 are shown as black, blue, green, and teal symbols, with the average spectrum for the flat surface shown as red diamonds. C4 and C12 are two different crystals on the beam.

Figure 6 shows emission spectra of the quantum dots in suspension and of quantum dots attached with polymer brushes to the flat silicon substrate adjacent to the photonic crystals. In suspension, the quantum dot emission maximum is observed at 9610 cm^–1^, which corresponds to a lead sulfide quantum dot diameter of about *d* = 3.46 nm.^82^ The width is about 1315 cm^–1^ full width at half-maximum, mostly due to size polydispersity,^83^ and the maximum intensity is nearly 100 counts/s. The spectra of the quantum dots attached to the silicon surface have a maximum near 8900 cm^–1^, which is red-shifted by about 710 cm^–1^ compared to the dots in suspension. The full width at half-maximum of the emission spectra on the surface is about 1070 cm^–1^, somewhat narrower than that in suspension.

We attribute the apparent shift and narrowing to two main qualitative features: first, the quantum dots experience different dielectric environments, where the dots in suspension are in water with an optical dielectric function of about ε_sus_ = 1.69.^84^ On the silicon–air surface, the dots experience a dielectric function ε_Si_ = 11.7 on the Si side and ε_air_ = 1 on the air side, which we interpret as an effective medium with , which is substantially greater than in suspension. In a high-epsilon environment, two-level emitters generally emit at a lower emission frequency due to the increased polarization of the environment by the emitting dipole,^85,86^ which qualitatively agrees with our observations in Figure 6. Second, in suspension the quantum dots are well separated, whereas on the flat silicon surface they are in close vicinity due to the densification from a 3D chemical reaction environment to a 2D surface. Consequently, we surmise that the dots on the surface experience energy transfer, including FRET,^85,87^ whereas the dots in suspension do not. In the case of energy transfer, high-frequency “blue-emitting” quantum dots effectively transfer their excitation to more red-emitting dots, hence the apparent enhancement of the low-frequency part of an emission spectrum at the expense of the high-frequency side. Such an enhancement of the red side and simultaneous decrease of the blue side of the spectrum agrees qualitatively with the observations in Figure 6.

Insights into effects of the employed brush surface chemistry are obtained by comparing the emitted intensity collected from different areas on the flat silicon surface by scanning the excitation laser spot across silicon beam 1, where the length scale of the probe is given by the focal diameter of the excitation laser (about 1 μm). The intensity maxima range from 83 to 139 cts/s, corresponding to less than 25% relative change from the average, which is a fairly small variation of absolute intensities, which indicates that the coverage with quantum dots is fairly homogeneous across the silicon surface. This micron-scale homogeneity is attributed to the covalent attachment of the quantum dots to the polymer brushes that generally leads to a homogeneous and well-controlled coverage. For comparison, on silicon beam 2 the quantum dots were not covalently attached to the polymer layer but physisorbed. Here, the intensities fluctuate much more while scanning the excitation laser across the sample, namely between 390 and 12,600 counts/s (more than 30 times), thus much more than above. We attribute the larger variations to aggregation of quantum dots in patches with few or even many dots clumped together.

Previously, we have probed the location of the quantum dots inside the photonic crystals with high spatial resolution (few tens of nanometers) by synchrotron X-ray fluorescence tomography,^55^ on the same samples as studied here, especially silicon beam 1. In the tomography study, we found that the detailed distribution of individual quantum dots shows some inhomogeneity along the length of each pore. However, averaged over the length of pores (several micrometers), the distribution of quantum dots is highly reproducible and homogeneous, which agrees with the optical probing above. From these observations combined, we conclude that the coupling strategy using the brush surface chemistry developed here is a successful one for silicon-based functional samples since the infiltration of the quantum dots into the photonic crystal nanostructures is homogeneous on optically relevant length scales.

**Figure 7:**
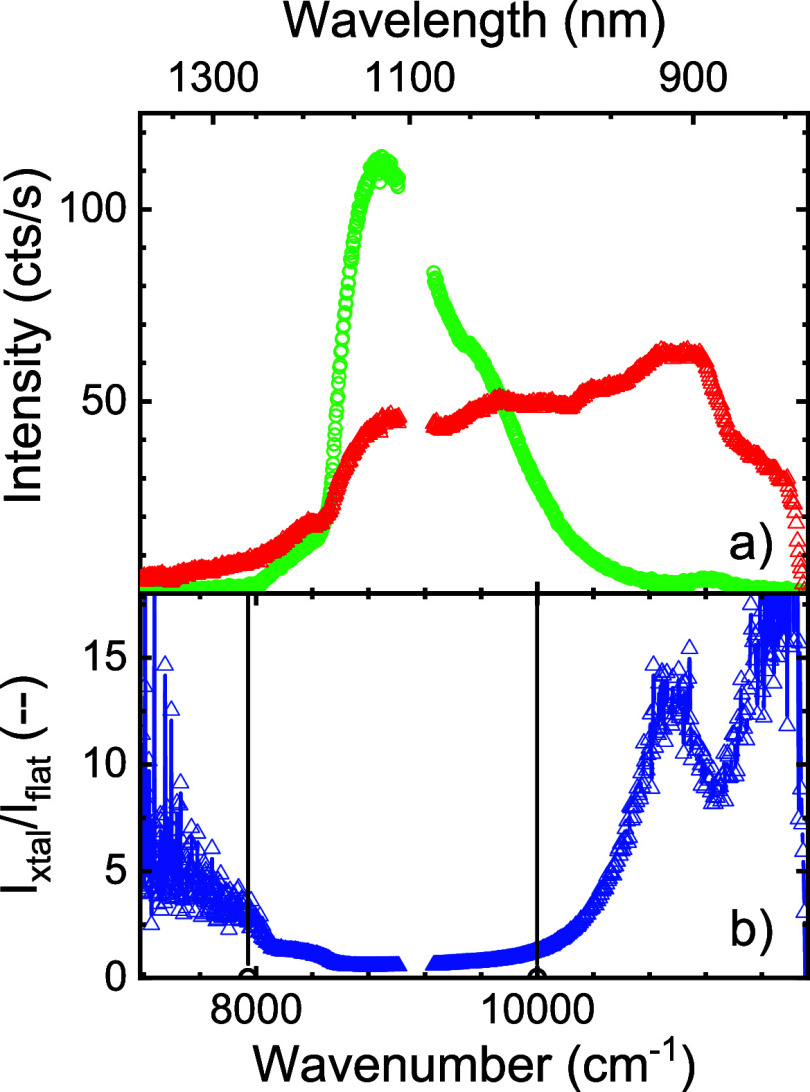
(a) Emitted intensity in counts/s versus photon energy (in wavenumbers, bottom abscissa) and wavelength (top abscissa) of quantum dots in crystal 12 (at *Y* = 0) on silicon beam 1 (red triangles). The green circles indicate the average spectrum of quantum dots on a flat silicon surface. (b) Ratio of the crystal spectrum *I*_xtal_ to reference spectrum on a flat surface *I*_flat_ (blue triangles). The estimated band gap edges are shown as vertical black lines.

Figure 7a shows the emission spectrum of the quantum dots infiltrated in a 3D photonic band gap crystal and for comparison the spectrum taken on a flat substrate, where the dots were in both cases attached by the same chemical procedure. The spectrum on the flat silicon surface has an emission maximum at 8900 cm^–1^ and a shoulder at 9600 cm^–1^, as shown in Figure 6. The spectrum of the quantum dots inside the photonic crystal is dramatically modified with respect to the spectrum on the silicon surface (and also compared to the suspension): the emission maximum has shifted to around 11,000 cm^–1^; in the frequency range of around 9000–10,000 cm^–1^ where quantum dots on Si (and in suspension) show emission maxima, the emission from inside the crystal is strongly inhibited. In addition to different spectral shapes, there are also other remarkable differences in the emitted signal: in the frequency window between 8500 and 9750 cm^–1^ the quantum dots in the crystal have a considerably lower count rate; in the low-frequency range below 8500 cm^–1^ the quantum dots in the crystal have a similar count rate as on the substrate, and above 9750 cm^–1^ the count rate is greater.

To discern the effect of the crystal on the emission spectrum, we show in Figure 7b the ratio of the silicon photonic crystal spectrum and the reference spectrum taken on the flat silicon substrate. This relative intensity spectrum shows a broad inhibition range between about 8000 and 10,000 cm^–1^ that agrees with the photonic band gap expected from the DOS calculations. At low photon energies, the relative intensity decreases with wavenumber increasing to the gap. At high photon energies above the gap, the relative intensity increases, showing a marked peak at 10,900 cm^–1^ that matches the peak in the DOS spectrum in Figure 2a, and a further increase to 11,600 cm^–1^. While the reference spectrum in Figure 7a has relatively low count rate in this range, the absolute counts are definitely systematic and significant since they were averaged for 600 s (see Supporting Information), thus corresponding to >10^3^ cts, which corresponds to a <0.5% relative error from Poisson statistics. The significance may be further appreciated from the fact that while the ratio in Figure 7b shows variations, these variations are much smaller than the overall frequency dependencies. The relative intensity in Figure 7b varies up to 15 in the peak at 10,800 cm^–1^, which differs from the relative intensity in the calculated relative DOS in Figure 2b that equals about 2.5 at the same peak. Several reasonable yet competing explanations for the overall difference in scale factor include: (i) a possible difference in areal density between quantum dots on the flat substrate versus those inside the curved pores, (ii) a possible difference in total number of quantum dots on the flat substrate versus inside the pores (due to different layer thickness), and (iii) a possible difference in optical collection efficiency between the flat substrate (well-defined focus) versus the photonic crystal where above-gap excitation light will be strongly scattered.^77^ Since it is at this time not possible to conclusively quantify these explanations, we scale each measured relative intensity spectrum by a single scale factor such that the peak at 10,800 cm^–1^ matches the calculated one, where the resulting factors are listed in Table S5 of Supporting Information.

**Figure 8:**
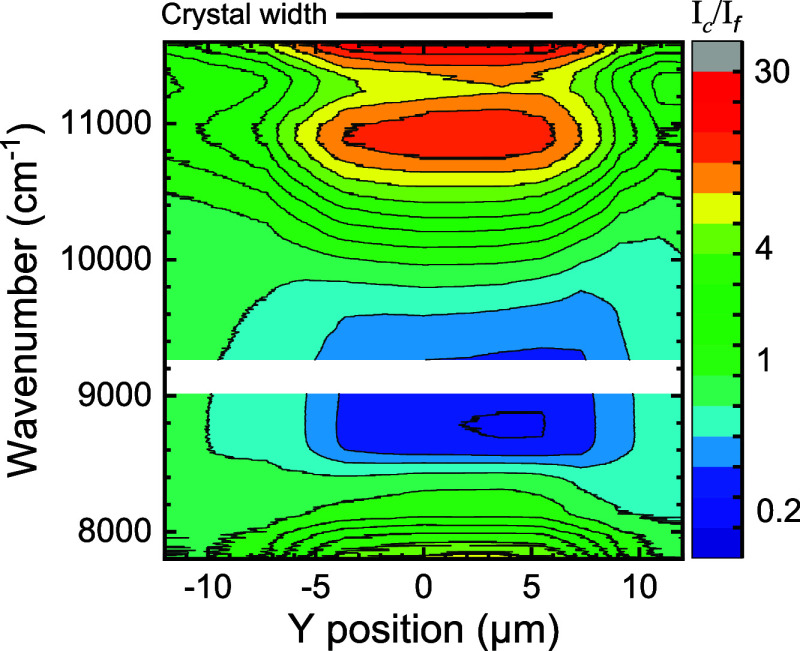
Spectral and *Y* position map of the emission inhibition for quantum dots in crystal 12 on silicon beam 1, obtained from spectra taken at 23 positions. The color map shows the inhibition defined as the ratio of a *Y*-dependent spectrum taken on the crystal and the flat Si surface reference spectrum (*I*_c_/*I*_f_). Green color (*I*_c_/*I*_f_ ≃ 1) indicates no inhibition, blue (*I*_c_/*I*_f_ < 1) is inhibited, and green, orange, and red (*I*_c_/*I*_f_ > 1) are enhanced. The size of the crystal is indicated at the top.

For the same photonic crystal 12 as probed in Figure 7, we performed a position-dependent scan (in the *Y* direction parallel to the crystal–air interface) to verify that the inhibited emission indeed correlates with the presence of the photonic band gap crystal. The ratio of the intensity of the quantum dots in and near the photonic band gap crystal to the intensity on the substrate far away from the crystal (*I*_c_/*I*_f_) is plotted in Figure 8 as a map versus the frequency and *Y* position. Far away from the crystal, this ratio closely equals 1 at all frequencies. The emission is observed to be most strongly inhibited at the center of the crystal at *Y* = 4 μm, in agreement with recent theoretical position-dependent calculations of the local density of states.^88^ The inhibition extends over 10 μm which is in agreement with the crystal extent shown in Figure 3b.

The inhibition smoothly decreases toward the edges of the photonic crystal, due to the finite size of the illuminating pulsed diode laser. In the same *Y* range where the crystal reveals inhibited emission, there is also a substantial enhanced emission near 11,000 cm^–1^, that matches with the theoretically predicted DOS peak at a reduced frequency ω̃ ≡ *a*/λ = 0.76 as shown in Figure 2a. At both higher wave numbers near 12,000 cm^–1^ and lower wave numbers near 7500 cm^–1^, there is additional enhanced emission, but due to the limited emission range of the quantum dots, no clear features can be identified that can be compared to the theoretical DOS.

From the major differences between the spectra collected from the crystal versus those on silicon, it is clear that the 3D photonic band gap crystal has a dramatic influence on the emission of the quantum dots. We discuss several possible hypotheses of why the quantum dots reveal a major inhibition in their emission spectra when they are placed inside a 3D photonic band gap crystal, namely, (1) a Franz–Keldysh effect, (2) FRET from quantum dots to the silicon, (3) FRET between the quantum dots, (4) hot-electron transfer from the quantum dots to the silicon backbone, (5) a preferential infiltration of small quantum dots into the crystal pores, and (6) the presence of a 3D photonic gap.

The first hypothesis proposes that the emitted intensity is reduced due to an increased optical absorption, due to a Franz–Keldysh effect whereby the quantum dot’s electron and hole wave functions increasingly “leak” into their electronic band gap.^86,89,90^ First, a strong electric field is necessary, which is absent in our experiments; second, the increased absorption reported in the literature^86^ is relatively weak and insufficient to explain a 5 to 30-times inhibition as described below; and third, the increased absorption occurs in the intrinsic absorption range of the quantum dots, which occurs at much higher photon energy than the photonic gap. Therefore, this hypothesis is unlikely, so we reject it.

The second hypothesis proposes that the quantum dots reveal FRET to the silicon backbone, as recently reported by Tabernig et al.^91^ First, the quantum dots are spaced from the silicon backbone by the polymer brush layer that has a thickness of 30–35 nm. While a quantum dot could possibly penetrate into the polymer brush layer, since we aimed for a high density polymer layer, it is unlikely that a quantum dot comes sufficiently close (1–10 nm) to the silicon surface to experience FRET. Moreover, the PbS quantum dots have a PEG shell that further shields FRET. Second, FRET is usually associated with a red shift of emission spectra,^85^ which is not apparent at all in Figure 7a, on the contrary, the spectra rather appear to show a blue shift for the crystal. Therefore, we find this hypothesis so unlikely that we reject it.

The third hypothesis proposes that the inhibition is caused by FRET between the quantum dots. FRET generally results in a red shift of the emission spectrum. First, if there would be more FRET in the crystal than on the flat substrate, the relative spectrum would reveal a monotonous negative trend, and if there would be less FRET in the crystal, the relative spectrum would reveal a monotonous positive trend, Second, the quantum dots have the same chemical environment in the crystals as on the flat reference substrate; hence, electron transfer seems equally likely on the reference samples, which corresponds to zero change in the relative intensity spectrum, in contrast to our observations. Neither of the three hypothesized spectral features match the observed inhibition; therefore, we reject this FRET hypothesis.

The fourth hypothesis proposes that highly excited “hot” electrons inside the quantum dots may be transferred to a semiconductor backbone, see, e.g., Tisdale et al.^92^ Since the quantum dots have a similar chemical environment in the crystals as on the substrate, electron transfer seems equally likely on both samples, which corresponds to zero change in the relative intensity spectrum, in contrast to the observed inhibition. Therefore, we reject this hypothesis.

The fifth hypothesis proposes that the smaller quantum dots among the whole population are preferentially infiltrated into the photonic crystal pores. First, since the crystal pores have a diameter greater than 260 nm, whereas the quantum dots have much smaller diameters of about 3 to 6 nm, it is unlikely that steric effects affect infiltration. Second, a preferential infiltration of smaller dots would lead to an effective blue shift of the crystal spectrum compared to the reference and thus to a relative spectrum with a monotonous positive trend, which does not match the observed inhibition; therefore, we reject this hypothesis.

Finally the sixth hypothesis proposes that the strong spectral inhibition is caused by a 3D photonic band gap in the crystal. Indeed a gap is designed to be present in such crystals, see, e.g., refs (76 and 93), and below we report further support for this hypothesis, namely that the gap systematically shifts when crystal properties (here, the pore radii) are varied. Therefore, we sustain this hypothesis.

### Comparison to Photonic DOS

While the observation of a broad and strong inhibition that corresponds to the expected complete 3D photonic band gap in the photonic DOS is at this point remarkable and exciting, it is also surprising in view of our initial expectation that the quantum dots bound to the polymer brushes would have a high quantum efficiency.

From previous studies,^21,94,95^ it is known that an important aspect in spontaneous emission control is the extent of the nonradiative decay (with rate Γ_nonrad_) of the quantum emitter in comparison to the desired radiative decay (with rate Γ_rad_). This extent is expressed by the quantum efficiency η that is equal to η ≡Γ_rad_/Γ_tot_, with Γ_tot_ being the total emission rate equal to the sum of the radiative and the nonradiative rates Γ_tot_ = Γ_rad_ + Γ_nonrad_. It turns out that these properties decide whether the LDOS is apparent in either continuous wave intensity spectra or time-resolved observations. From the rate equation, the continuous wave emitted intensity *I*(ω) (with spectra as in Figure 7 above) is derived to be equal to^94^
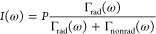
2where *P* is the pump rate or excitation rate.^d^ If emitters have a high quantum efficiency near unity (η ≃ 1) and hence Γ_rad_ ≫Γ_nonrad_, it follows from eq 2 that nearly every incident pump photon is converted into an emitted photon (*I* ≃ *P*), hence the emitted intensity is independent of the LDOS. Conversely, if the emitters have a low quantum efficiency (η ≪ 1, hence Γ_rad_ ≪ Γ_nonrad_), it follows from eq 2 that the intensity *I*(ω) is equal to
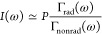
3which is proportional to the radiative rate and thus to the LDOS, and this is the regime of our observations here.

**Figure 9:**
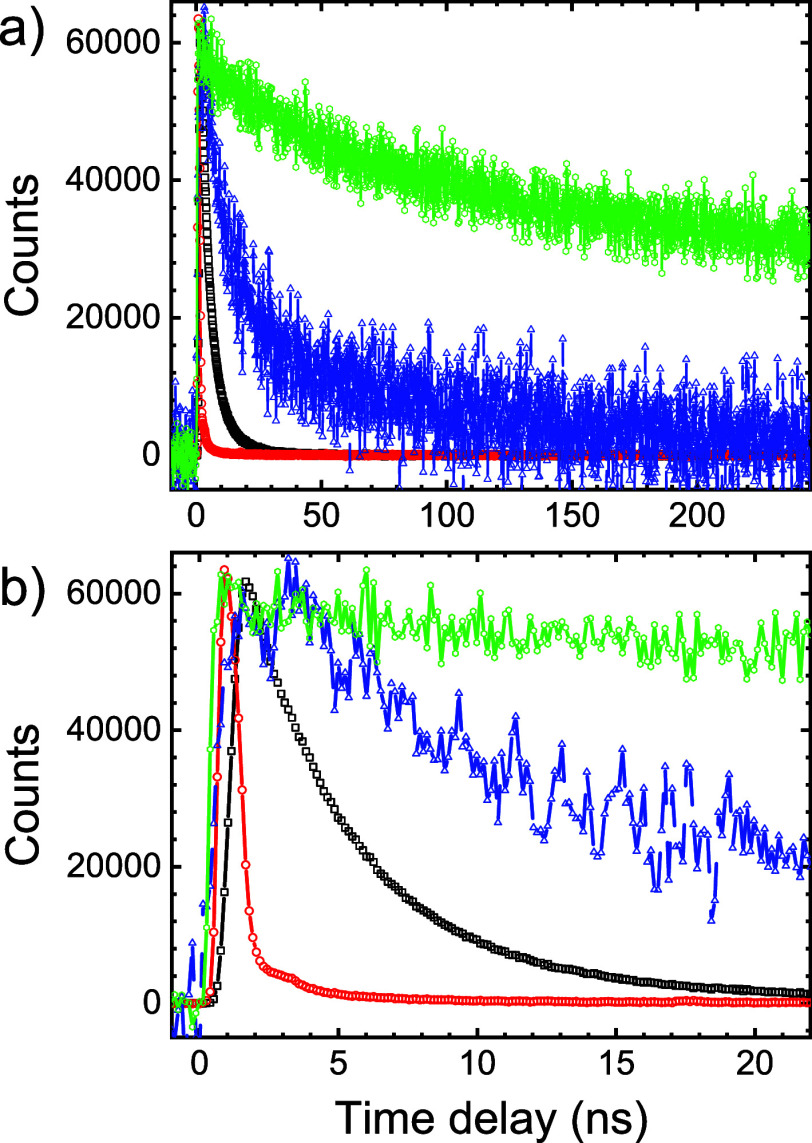
Time-correlated single photon counting of PbS quantum dots on a flat substrate on silicon beam 1 (black squares), in crystal 4 (blue triangles), in crystal 12 (red circles), and in suspension (green hexagons, at 1090 nm). Counts per bin are plotted versus photon arrival time. (a) Expanded time scale showing the slow decay in suspension and crystal 4 and (b) zoomed-in scale to show the fast decay on the flat substrate and in crystal 12.

To assess whether the quantum dots in our experiment are in the high- or low-efficiency limits, let us consider their microscopic placement in the various samples, illustrated in Figure 4, and the time-resolved emission shown in Figure 9. In the suspension, the quantum dots have low density (see Figure 4a) and are manufactured to have as high a quantum efficiency as possible. In time-resolved emission in Figure 9, the quantum dots in suspension show a decay with an emission rate of Γ_tot_ = 0.223 ± 0.004 μs ^–1^, which matches well with literature results on good quality PbS quantum dots.^82,96,97^

When the quantum dots are bound to the silicon substrate or inside the silicon photonic crystal (see Figure 4b,c), it is reasonable that their local number density is much greater since during the preparation the quantum dots are concentrated from volume to surfaces. Therefore, we expect the quantum dot-quantum dot quenching to be greater, corresponding to a greater nonradiative rate. Moreover, during the preparation the quantum dots are exposed to Cu catalysts that also increase the nonradiative rate.^98,99^ Indeed, in time-resolved emission in Figure 9, we observe that the quantum dots inside the 3D photonic crystals and on the substrate show much faster decay than that in suspension. On the Si substrate, we find decay rates between Γ_tot_ = 190.3 ± 0.6 and Γ_tot_ = 267 ± 0.6 μs ^–1^, and in the photonic crystal, Γ_tot_ = 153.0 ± 1.3 μs ^–1^, see all data compiled in Supporting Information, Table S4. In other words, the decay rates in and on silicon are about 850 to 1200-fold greater than in suspension, due to a similar major increase of the nonradiative decay rate Γ_nonrad_.

**Figure 10:**
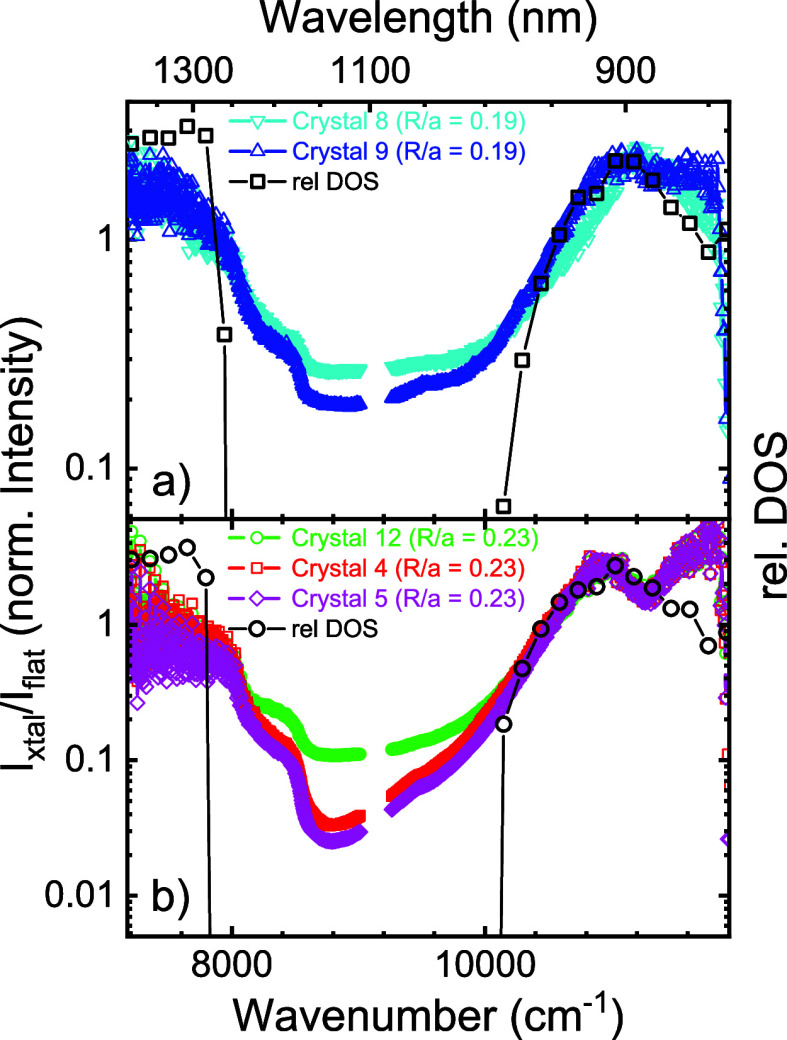
Normalized emission spectra of 3D photonic crystals on silicon beam 1: (a) crystal 9 (blue upward triangles), crystal 8 (teal downward triangles) and (b) crystal 4 (red squares), crystal 12 (green circles), and crystal 5 (magenta diamonds). The ratio of the spectra measured on the structures and the flat reference spectrum is plotted versus photon energy in wavenumbers and wavelength (top abscissa). Connected black open symbols are the theoretically calculated relative DOS, for relative pore radii (a) *R*/*a* = 0.19 and (b) *R*/*a* = 0.23.

While one might optimally speculate that a much faster decay (with an increased decay rate) is caused by an increased DOS, we reject this hypothesis since the fast decay occurs both in the photonic crystal (major DOS modifications; see Figure 10) and on the Si substrate where DOS modifications are minor. Since in both cases the nonradiative decay rate is so large, and since this rate is insensitive to the very different photonic DOS of these two different samples, it is reasonable that both samples reveal a similar fast decay. Moreover, the increased total emission rates (see Table S4) anticorrelate with the decreasing intensities (see Figure 6), which is indicative of quenching. Therefore, we conclude from these observations and from the physicochemical considerations that the total decay rate Γ_tot_ of the quantum dots on Si and in the photonic crystals is dominated by the nonradiative decay rate Γ_nonrad_. Therefore, we conclude that the emission spectra as shown in Figure 7 are in the low quantum efficiency limit, and hence, the observed continuous wave intensity spectra are proportional to the DOS, which we now pursue.

### Emission Spectra in Photonic Band Gap Crystals

Figure 10 shows the relative emission spectra of the PbS quantum dots infiltrated in the 3D silicon photonic band gap crystals with two different sets of pore radii, namely, in panel (a) crystals with radii *R* = 129 nm (reduced radii *R*/*a* = 0.19) and in panel (b) with radii *R* = 156 nm (reduced radii *R*/*a* = 0.23). In this section, we use a two-step normalization. On one hand, the emission spectra of quantum dots in the crystals are referenced to spectra collected on flat substrates (see Figure 7), to normalize out the intrinsic spectrum of the quantum dots and thus concentrate on the spectral shape of the (normalized) local density of states of the crystals. Since the number of quantum dots and the collection efficiencies differ between the photonic crystals and the flat substrates, the count rates between these measurements differ substantially, hence we can unfortunately not calibrate the number of quantum dots between both situations. The second step is to normalize relative spectra as in Figure 7 to theory as in Figure 2b. An ideal reference is a photonic crystal with a small lattice parameter such that emission occurs in the low-frequency limit below the gap, where such a crystal behaves as an effective homogeneous medium, see Figure 2a and previous ref (94). Since such crystals were not available during our study (due to the complexity of fabrication, preparation, and X-ray characterization), we propose to use the flat silicon substrate as an effective-medium reference sample since it also has a parabolic DOS and a well-defined effective ϵ (see subsection “Emission spectra”). In the thus calibrated relative emission spectra (see Figures 7b and 10), we observe the same characteristic features as in the theoretical spectra, namely, the clear peak at 10,900 cm^–1^ and of course the major inhibition range. Hence, a relative intensity of 14 in the peak in Figure 7 is normalized to correspond to a relative DOS of 3 in Figure 2b. All resulting normalization constants are listed in Table S3.

In Figure 10a, we see that the relative intensities for two different crystals with pore radii *R* = 129 nm reveal a strong inhibition between about 8000 and 10,200 cm^–1^, in very good mutual agreement. The very good reproducibility confirms that both the photonic crystal fabrication and the quantum dot infiltration methods are also well reproduced. The minimum relative emission near 8800 cm^–1^ is about 0.2 to 0.25, corresponding to maximum inhibitions in the photonic band gap of 4 to 5-fold, relative to the reference situation described above. The theoretical DOS has a gap between 8000 and 10,200 cm^–1^, in very good agreement with the observations. Indeed, the (relative) pore radii pertaining to the calculations *R*/*a* = 0.19 agree well with the pore radii derived from the SEM images and the radii used in the sample design.

Figure 10b shows the relative intensities for three other crystals that all have larger pore radii *R* = 320 nm in comparison to the crystals in panel (a). All three crystals reveal a strong inhibition between about 8000 and 10,400 cm^–1^, in very good mutual agreement. The inhibition of one crystal (green) amounts to about 10-fold, whereas the other two reveal striking inhibitions up to 30-fold. In view of possible systematic experimental errors such as alignment, we estimate the overall inhibition to be 20 ± 10-fold. The greater inhibition observed for the second set of crystals [in panel (b)] compared to the first makes intuitive sense since the observed inhibition gap is wider. Moreover, the pore radii of the second set of crystals are closer to the value *R*/*a* = 0.245 that is known to reveal the broadest 3D photonic band gaps in silicon inverse woodpile photonic crystals.^75,100^

Compared to the theoretical DOS, we find that the observed enhancement peak near 11,000 cm^–1^ in Figure 10 matches well in width and shape. A limitation to our interpretation is obviously the use of theory for infinite and perfect crystals, as it predicts zero DOS in the gap that appears to match fairly well on a linear scale as in Figure 2, whereas it appears dramatic on a semilog scale as in Figure 10. Similarly, the theoretical DOS just below the gap is higher with a sharp edge, as opposed to the lower and smoother observed edge; the difference is tentatively attributed to fabrication imperfections that are difficult to model. Nevertheless, the theoretical DOS reveals a gap between 7900 and 10,200 cm^–1^, which agrees very well with our observations.

## Discussion and Conclusions

The inhibition in 3D inverse woodpile crystals was previously studied by Leistikow et al. in time-resolved studies on high quantum efficiency quantum dots randomly infiltrated throughout the pores.^31^ Leistikow et al. observed an inhibition of about 10-fold, which agrees remarkably well with our present results. We speculate that this agreement is coincidental in view of several notable differences between both studies.

First, in the present study, the quantum dots are positioned by the polymer brushes to a limited set of positions in the unit cell (near the axes of the pores, see Figure 3b), whereas Leistikow et al. infiltrated the quantum dots as a suspension in the pores; hence, their quantum dots sample all spatial positions in the pores and thus many more positions in the unit cell, namely, about 80% of the whole volume. From theory, it is known that the LDOS at a single position varies much more strongly with frequency (thus yielding more inhibition) than when such the LDOS is averaged over many more positions inside a photonic crystal’s unit cell. In the extreme case of averaging over all positions, LDOS becomes the DOS, which yields a smoother result, see refs (69 and 88).

Second, here we study time-averaged emission of low-efficiency quantum dots whereas Leistikow et al. studied time-resolved emission of high-efficiency dots; the second technique allows to distinguish between different dynamics in a population of quantum emitters (see van Driel et al.^101^), whereas a time-average study reveals an average emission rate. Within a distribution of emission rates that is accessible by time-resolved studies, one may thus find more variable emission rates, whereas averaged rates are usually much smoother.

A third notable difference is that Leistikow et al. kept their quantum dots in (toluene) suspension, as a result of which the refractive index ratio with the silicon backbone is less, hence the photonic band gap is narrower and hence the inhibition is reduced. In the present study, the quantum dots are attached to the brush polymers inside the pores. It is speculated that the overall density of material inside the pores (see the schematic cross section in Figure 3) also increases the dielectric function ϵ inside the pores. This hypothesis is inspired by the observation in our previous X-ray imaging study that tomography (by elastic scattered X-rays) was not feasible due to too little contrast in electron density between the silicon backbone and the filled pores.^55^ In summary, compared to the work of Leistikow et al., the reasons above could respectively result in increased, decreased, and similar decay rates; hence, the similar observed inhibition seems coincidental to us.

The inhibition of quantum emitters was also studied on completely different 3D photonic crystals.^25,26,30,102^ In TiO_2_ inverse opal photonic crystals made by self-assembly,^103^ Koenderink et al. observed a broad-band angle-integrated inhibition in the emission spectra of laser dye by 5-fold.^25,94^ The inhibition is likely less than observed here in view of the fact that TiO_2_ photonic crystals have a lower refractive index contrast than our Si nanostructures, combined with the feature that these inverse opals do not possess a full 3D band gap as opposed to our photonic crystals. In GaAs woodpile photonic crystals, Ogawa et al. reported an inhibition of 45-fold on embedded quantum wells.^26^ While the index contrast of GaAs nanostructures is nearly the same as in our Si crystals, the inhibition is slightly larger than observed here, which we attribute to the feature that their emitters are only located in the central layer of the woodpile structure and thus well-shielded, whereas in the present case, the emitters are located over the whole length of the pores and thus also near the crystal surface where they are less shielded from the vacuum. Li et al. studied PbS quantum dots in polymer photonic crystals and reported an inhibition of 20%.^102^ It is reasonable that this inhibition is less than that observed here in view of the lower index contrast and concomitant absence of a full photonic band gap. Jorgensen et al. studied quantum dots in TiO_2_ photonic crystals and observed inhibitions of about 4-fold,^30^ which is also reasonably less than our observations in view of the lower index contrast and concomitant absence of a full photonic band gap. Taken together, all results confirm the long-standing expectation that substantial control of emission of embedded quantum emitters requires nanostructures with high-index semiconductors as the backbone in order to have sufficient refractive index contrast and preferably even a full 3D band gap.

As future extensions of our current study, it will first be relevant to calculate the LDOS for the positions where we detected the quantum dots to reside using X-ray imaging. Since it is therefore necessary to introduce X-ray imaging data into numerical ab initio computations of Maxwell’s equations, it will be relevant to extend the recent method by van Willenswaard et al., who describe a computational framework to introduce X-ray imaging data into computations.^104^ Second, to allow for time-resolved studies with quantum dots positioned by brush polymers, it will be important to avoid the steps that induce quenching of the quantum efficiency of the dots. The most important step will be to avoid the copper catalysts, for instance, by invoking copper-free ATRP.^105^ In addition, it will be relevant to design strategies to shield the quantum dots from oxygen and water that are also well-known quenchers of highly efficient quantum dots, see, e.g., the experimental details in refs (27 and 51). Third, the presence of brush polymers offers in future a very exciting prospect, namely to employ the length actuation of the brushes by physicochemical means.^106−108^ As a result, it may be feasible to tune or even switch the LDOS, the emission rate, and inhibition by actuating the brushes. This in turn opens prospects to employing such actuated quantum emitters as sensitive positions or pH-sensors or as novel platforms for tunable (photo)-chemistry.

## Supporting Information Available

Scheme of the chemical synthesis; overview of samples and crystals studied; schematic of the optical setup; overview of emission rates; and normalization factors to scale relative intensity spectra. A preprint of this manuscript is available on the ChemRxiv preprint server^109^ (PDF)

## Footnotes

a It is well-known that vacuum fluctuations contribute only one-half of the emission rate, the other half being induced by electrodynamical back action, see refs (15 and 21).

b The noise-like features, especially in the low-frequency region, are the result of binning in frequency space in the process of mode counting. Hence, these features are in fact finite-size speckles.

c We express frequency as a reduced frequency ω̃ = ωa/(2πc)), with ω the frequency, *a* the lattice parameter, and *c* the speed of light (not to be confused with the lattice parameter in the *X*, *Z* directions). The reduced frequency ω̃ also equals (*a*/λ) which is convenient in the interpretation of experiments.

d Since the excitation occurs at a different (much higher) frequency, it is independent of the emission frequency ω.
