# Supplementary material for: Strongly Inhibited Spontaneous Emission of PbS Quantum Dots Covalently Bound to 3D Silicon Photonic Band Gap Crystals
Source: J Phys Chem C Nanomater Interfaces. 2024 May 28;128(22):9142–53. doi: 10.1021/acs.jpcc.4c01541 (PMC11163470; doi:10.1021/acs.jpcc.4c01541)
Supplement: Supplementary file 1 — jp4c01541_si_001.pdf [file jp4c01541_si_001.pdf]

Supporting information for: Strongly Inhibited Spontaneous Emission of PbS Quantum Dots Covalently Bound to 3D Silicon Photonic Band Gap Crystals

Andreas S. Schulz,[†,‡,¶] Marek Kozoň,[†,§] G. Julius Vancso,[¶,‖] Jurriaan Huskens,[‡] and Willem L. Vos[*,†]

†*Complex Photonic Systems (COPS), MESA+ Institute, University of Twente, P.O. Box 217, 7500 AE Enschede, The Netherlands*

‡*Molecular Nanofabrication (MNF), MESA+ Institute, University of Twente, P.O. Box 217, 7500 AE Enschede, The Netherlands*

¶*Materials Science and Technology of Polymers (MTP), MESA+ Institute, University of Twente, P.O. Box 217, 7500 AE Enschede, The Netherlands*

§*Mathematics of Computational Science (MACS), MESA+ Institute, University of Twente, P.O. Box 217, 7500 AE Enschede, The Netherlands*

‖*Sustainable Polymer Chemistry (SPC), MESA+ Institute, University of Twente, P.O. Box 217, 7500 AE Enschede, The Netherlands*

E-mail: w.l.vos@utwente.nl

Supporting Information Available

This supplementary features 1) details of the polymer brush chemistry and quantum dots, 2) an overview of the photonic crystals that were studied, 3) details of the optical setup, 4) an overview of all emission rates. 5) an overview of all normalization factors.

1 Polymer brush chemistry and quantum dots

The chemical synthesis is described in three parts: firstly, the attachment of the initiator to the silicon beam, secondly, the SI-ATRP of the poly(glycidyl methacrylate) brushes, and thirdly the coupling of the quantum dots, see scheme S1 below.

Scheme S1 - Chemical synthesis:

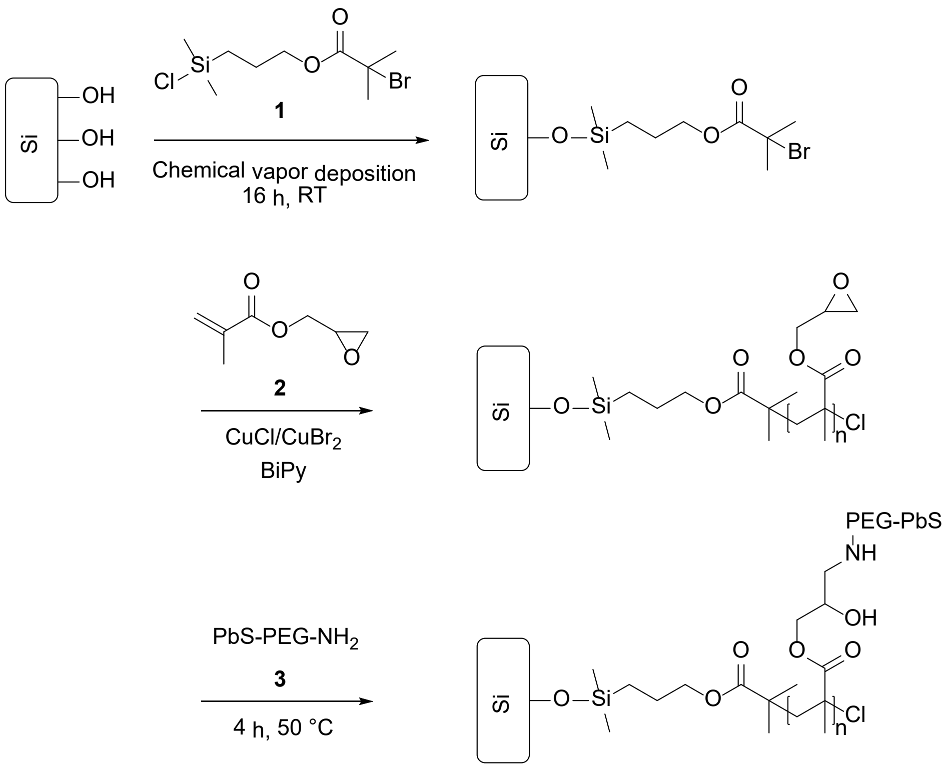

1.1 Chemical vapor deposition of the initiator

The ATRP initiator was synthesized following Ramakrishnan *et al.*[S1] A piranha solution was prepared with the ratio of 3 parts H_2SO_4 to 1 part H_2O_2 (volume ratio, v/v%). The silicon beam was placed into the sample holder. The sample holder was lowered gently into the piranha solution and the sample was cleaned for 30 min. The silicon beam was rinsed 10 times with water and ethanol. A desiccator was rinsed by vacuum pumping for 5 to 10 min. The initiator was taken from a vial using a argon purged syringe. A few droplets of initiator (30 μL) were placed inside a small plastic petri dish that was placed in the middle of the desiccator. The Si beam was placed close to the petri dish such that the vapor diffuses into the pores of the photonic crystal. The desiccator was vacuum pumped for 15 min, and the chemical vapor deposition (CVD) process proceeded for 16 h. Next, the beam was placed into a flask with 100 mL toluene in an ultrasonication bath for 20 s to remove excess initiator. Each substrate was rinsed 10 times with ethanol and water before the substrates were dried under a nitrogen stream. The silicon beam was stored in a nitrogen box.

1.2 Chemical synthesis of PGMA polymer brushes

Glycidyl methacrylate (GMA, 10 mL, 75 mmol) monomer and 2,2‘-bipyridine (BiPy, 282 mg, 1.81 mmol) were added to a mixture of water (2 mL) and methanol (8 mL).[S2] The solution was purged with argon for 30 min under continuous stirring. CuCl (72.8 mg, 0.74 mmol) and $CuBr_2$ (7.8 mg, 0.035 mmol) were added to another flask and flushed with argon for 15 min. The monomer solution was transferred by a previously flushed syringe to the catalyst flask. The resulting polymerization solution was stirred for an additional 30 min under argon. The silicon beam was taken out of the nitrogen box shortly before the reaction and was placed into a reaction flask. The reaction flask was attached to a vacuum pump. Afterwards the monomer solution was transferred to the reaction flask containing the silicon beam. The polymerization was allowed to proceed for 60 min. The silicon beam was cleaned extensively with acetone, water-free dimethyl sulfoxide, and 0.1 M ethylene-

diaminetetraacetic acid (EDTA) solution to remove non-reacted monomer, metal complex, and remaining catalyst residues. The silicon beam was dried in a nitrogen stream to obtain the sample with the thin poly(glycidyl methacrylate) (PGMA) brush film.

1.3 Covalent attachment of the quantum dots to the polymer brush layer

In the last step the quantum dots are introduced. The inorganic lead sulfide core of the quantum dots is covered by a PEG-NH_2 ligand that is used to covalently attach the QDs to the PGMA layer on the silicon photonic crystal.

2 Photonic crystals studied

Table S1 lists the silicon bar samples and photonic crystals studied here.

Table S1 - Overview of samples and crystals studied:

Sample name in text	Sample name in lab	Remarks
**Silicon beam 1**	PG-EBAR-30032017-CH4B	Si beam with 14 photonic crystals fabricated with the single-step etch mask process see Ref.,[S3] with crystals:<br>C1: 2D, d = 260 nm, no cavity (nc);<br>C2: 2D, d = 230 nm, nc;<br>C3: 2D, d = 230 nm, nc;<br>C4: 3D, d = 320 nm, with cavities;<br>C5: 3D, d = 320 nm, nc;<br>C6: reference structure;<br>C7: 2D, d = 260 nm, nc;<br>C8: 3D, d = 260 nm, nc;<br>C9: 3D, d = 260 nm, nc;<br>C11: 3D, d = 320 nm, nc;<br>C12: 3D, d = 320 nm, nc;<br>C13: 2D, d = 320 nm, nc;<br>C14: 2D, d = 320 nm, nc;<br>C15: 3 × 2D, d = 260 nm, nc.
**Silicon beam 2**	PG-EBAR-30032017-CH3B	Silicon beam with 5 photonic crystals fabricated with the single-step etch mask process described in Ref.[S3,S4] The silicon bar is coated with a thin polymer brush layer of poly(glycidyl methacrylate). PbS-OA quantum dots are dip-coated onto the sample. Crystals:<br>C1: 3D, d = 320 nm, nc;<br>C2: 3D, d = 260 nm, nc;<br>C3: 3D, d = 260 nm, nc;<br>C4: 2D, d = 260 nm, nc;<br>C5: 3 × 2D, d = 320 nm, nc.

Sample silicon beam 1 consists of six 3D and eight 2D photonic crystals, with lattice parameters either a = 680 nm or a = 686 nm, and c = 481 nm. Here, we present measurements on crystals C4 (3D with cavities, pore diameter $d = 320$ nm), C5 (3D, no cavity, $d = 320$ nm), C8 (3D, no cavity, $d = 260$ nm), C9 (3D, no cavity, $d = 260$ nm) and C12 (3D, no cavity, $d = 320$ nm). Sample silicon beam 2 consists of six 3D and eight 2D photonic

crystals, all with lattice parameters $a = 680$ nm or $a = 686$ nm, and $c = 481$ nm. Here, we present one measurement on crystal C1 (3D, no cavity, pore diameter $d = 320$ nm).

3 Optical setup

We present here the optical setup used to collect emission from near-IR emitting quantum dots inside 3D silicon photonic band gap crystals, see Figure S1 below. Earlier versions of the setup have been reported in Refs.[S5–S7]

Figure S1 - Schematic optical setup:

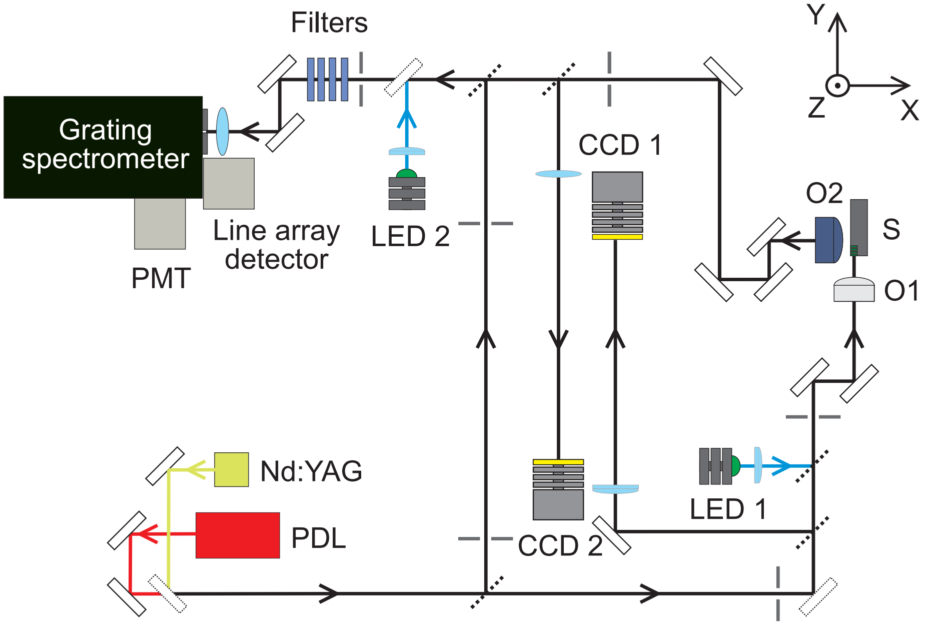

To excite the quantum dots, we used a pulsed diode laser (PDL, Picoquant: LDH-C 690) emitting at $\lambda = 690$ nm, controlled by a driver (Picoquant: PDL 800-D) with a tunable repetition rate set to 62.5 kHz. The cw power of the pulsed diode laser was measured to be 9.71 mW within the single exciton regime. The lead sulfide quantum dots reside inside the 3D silicon photonic crystal (S) sample that is placed on a three-dimensional piezo stage to align and scan the position of the photonic crystal with respect to the objectives. For time-resolved measurements the sample is excited from the YZ-surface by focusing the incident

laser beam with objective O1 (numerical aperture NA = 0.12). Light emitted by quantum dots in the photonic crystal is collected from the XY-face by a high-NA objective O2 (NA = 0.72, Nikon CFIL Plan EPI SLWD). Two separate LEDs and CCDs are used to align both incident and detection foci on the front-face and the side-face of the photonic crystals. All mirrors are silver-coated to provide a high reflectivity in the near-infrared and the visible wavelength ranges.

Light collected from the quantum dots in the photonic crystal is filtered with two long-pass filters (Thorlabs KG850 and FEL1100) to remove stray laser light, sent to a grating monochromator (Princeton Instruments, Spectra Pro 2558, gold-coated optics). The monochromator has a turret with two gratings (900 g/mm Holographic for high resolution and 85 g/mm, blazed for 1.35 μm for low resolution), and a gold mirror to collect all light on one pixel. To record emission spectra, the output of the monochromator is directed to a liquid nitrogen cooled InGaAs line diode array (OMA-V: 1024-2.2 LN). The emission spectra were collected over 600 s integration time.

For time-correlated single photon counting experiments,[S8] the output of the monochromator is directed to a Peltier-cooled NIR photomultiplier tube (PMT, Hamamatsu H10330-75), equipped with a timing module (Picoquant: PicoHarp 300 counting module). The time-dependent lifetime measurements are integrated over 7200 s duration with a time-bin resolution of 288 ps.

The alignment of the setup is done in three steps: Firstly, a He-Ne laser beam is sent through the holder of the collecting objective O2, by reflecting the incident laser beam with a mirror to align onto the closed entrance slit of the spectrometer. Secondly, the excitation beam from the pulsed laser is aligned and focused onto the YZ surface of the silicon beam. As confirmation, the focus of the laser light is viewed by a CCD camera that is focused onto the same side of the Si beam. The position of the focus spot is chosen such that the laser hits the center of the silicon photonic crystal. Thirdly, the second, XY-crystal surface is aligned in the same way as the first surface by sending the excitation light beam on that

face. Since the emission of the near-infrared quantum dots is relatively weak, the signal is at first maximized by using the mirror in the monochromator to collect all counts to one pixel, see also Ref.[S5]

4 Overview of emission rates and scale factors

Table S2 lists the results of the time-resolved emission measurements on silicon beams 1 and 2 and on a coated silicon bar.

Table S2 - Overview emission rates:

measurement location	Γ_{mf} in μs $^{-1}$	$\Delta\Gamma$	χ^2_{red}
**silicon beam 1**			
QD suspension	0.223 ± 0.004	1.09(3)	1.083
flat: 50 μm above C12	190.3 ± 0.6	290 ± 4	1.087
flat: 75 μm above C12	267.0 ± 0.6	348 ± 4	1.171
flat: 100 μm above C12	258.4 ± 0.6	336 ± 4	1.136
C3 (2D)	257.4 ± 1.6	527 ± 11	1.024
C4 (3D)	173.0 ± 2.8	1200 ± 300	1.047
C5 (3D)	177.0 ± 2.1	1080 ± 230	1.108
C12 (3D)	153.0 ± 1.3	780 ± 130	1.070
C4 (3D), exception	10.6 ± 0.2	67 ± 22	1.057
**silicon beam 2**			
QD suspension	1.0175 ± 0.0004	0.0(7)	1.019
QD-cluster	1.34 ± 0.13	13.6 ± 2.1	1.155
flat: 30 μm above C1	8.18 ± 0.05	117.3 ± 2.4	1.233
flat: 50 μm below C4	11.929 ± 0.017	96.1 ± 0.5	1.706
flat: 70 μm below C4	11.771 ± 0.019	109.0 ± 0.7	1.821
C1 (3D)	70 ± 40	700 ± 700	0.987
**MK-Nano PbS**			
QD suspension	1.1280 ± 0.0008	0.314 ± 0.015	1.003
MK-Nano dip-coated	11.67 ± 0.05	16.17 ± 0.14	1.015

All histograms are analyzed with a log-normal distribution,[S9,S10] where Γ_{mf} is the most frequent decay rate, $\Delta\Gamma$ the width of the log-normal distribution, and χ^2_{red} is the reduced goodness-of-fit.

4.1 Scale factors of the relative intensity spectra

Table S3 - Normalization factors: Table S3 lists the normalization factors to scale each

Structure name	Normalization factor
**C8**	2.1
**C9**	2.3
**C12**	5.0
**C4**	16.7
**C5**	27.9

measured relative intensity spectra such that the peak at 10800 cm^{-1} matches the calculated one in the corresponding DOS spectrum (see Figure 2 in the main manuscript.)

A limitation to our calibration procedure is of course that the theoretical results pertain to an infinite crystal, hence the inhibition is perfectly 0 and the gap has sharp bounds, whereas the observed inhibition is rounded and finite. Based on the variations in the spectra, we estimate the error margins of the scale factors above, and the concomitant relative intensities, to be about 10% relative error.

References

(S1) Ramakrishnan, A.; Dhamodharan, R.; Rühe, J. Controlled Growth of PMMA Brushes on Silicon Surfaces at Room Temperature. *Macromol. Rapid Commun.* **2002**, *23*, 612–616.

(S2) Edmondson, S.; Huck, W. T. S. Controlled growth and subsequent chemical modification of poly(glycidyl methacrylate) brushes on silicon wafers. *Journal of Materials Chemistry* **2004**, *14*, 730–734.

(S3) Grishina, D. A.; Harteveld, C. A. M.; Woldering, L. A.; Vos, W. L. Method for making a single-step etch mask for 3D monolithic nanostructures. *Nanotechnology* **2015**, *26*, 505302.

(S4) Grishina, D. A. 3D silicon nanophotonics. *PhD thesis, University of Twente* **2017**,

(S5) Husken, B. H. Spontaneous emission of near-infrared quantum dots controlled with photonic crystals. *PhD thesis, University of Twente* **2009**,

(S6) Leistikow, M. D.; Mosk, A. P.; Yeganegi, E.; Huisman, S. R.; Lagendijk, A.; Vos, W. L. Inhibited Spontaneous Emission of Quantum Dots Observed in a 3D Photonic Band Gap. *Phys. Rev. Lett.* **2011**, *107*, 193903: 1–5.

(S7) Yeganegi, E. Controlling emission and propagation of light with photonic band gap crystals. *PhD thesis, University of Twente* **2014**,

(S8) Becker, W. *Advanced time-correlated single photon counting techniques*; Springer, Berlin, 2005.

(S9) Van Driel, A. F.; Nikolaev, I. S.; Vergeer, P.; Lodahl, P.; Vanmaekelbergh, D.; Vos, W. L. Statistical analysis of time-resolved emission from ensembles of semiconductor quantum dots: Interpretation of exponential decay models. *Phys. Rev. B* **2007**, *75*, 035329.

(S10) Nikolaev, I. S.; Lodahl, P.; van Driel, A. F.; Koenderink, A. F.; Vos, W. L. Strongly nonexponential time-resolved fluorescence of quantum-dot ensembles in three-dimensional photonic crystals. *Phys. Rev. B* **2007**, *75*, 115302.

This material is available free of charge via the Internet at `http://pubs.acs.org/`.
